# The Role of Type 1 Interferon in Systemic Sclerosis

**DOI:** 10.3389/fimmu.2013.00266

**Published:** 2013-09-06

**Authors:** Minghua Wu, Shervin Assassi

**Affiliations:** ^1^Division of Rheumatology and Clinical Immunogenetics, Department of Internal Medicine, University of Texas Health Science Center at Houston, Houston, TX, USA

**Keywords:** systemic sclerosis, innate immunity, type 1 IFN, interferon regulatory factor, IFN-inducible cytokines and chemokines

## Abstract

Systemic Sclerosis (Scleroderma, SSc) is an autoimmune disease characterized by vasculopathy, inflammation, and fibrosis that can lead to loss of organ function. Type I interferons (IFNs) are family of cytokines that mitigate the deleterious effects of viral and bacterial infections in the innate immunity system. Past several years, research efforts have been focused on the role of type I IFN and IFN-inducible genes in the pathogenesis of SSc. Polymorphisms in the Interferon regulatory factor (*IRF*)*-5*, *IRF7*, and *IRF8* are associated with SSc, Similarly, polymorphism of Signal Transducer and Activator of Transcription (STAT)-4, has been established as a genetic risk factor of SSc. IRFs and STAT4 proteins are key activators of type I IFN signaling pathways. An IFN signature (increased expression and activation of IFN-regulated genes) has been observed in the peripheral blood and skin biopsy samples of patients with SSc. Furthermore, a plasma IFN-inducible chemokine score correlated with markers of disease severity and autoantibody subtypes in SSc. In this review, we summarize our current knowledge of the role of type I IFNs and IFN-inducible genes in the pathogenesis of SSc and their potential role as biomarkers and therapeutic targets.

## Introduction

Systemic sclerosis (Scleroderma, SSc) is characterized by immune dysregulation, fibrosis, and vasculopathy although its pathogenesis is not completely understood (1). Disease morbidity and mortality remain high (2, 3). There is no definite cure for SSc and the available treatments have limited efficacy. The major hurdle in developing effective therapies for SSc is an incomplete understanding of disease pathogenesis. A better understanding of SSc pathogenesis is important for identifying more targeted and effective therapeutic approaches.

Recently, there has been an increasing interest in the role of type I interferons (IFNs) in pathogenesis and severity of SSc. IFNs are a heterogeneous family of multifunctional cytokines. They were originally identified as proteins responsible for induction of cellular resistance to viral infections. Type I IFNs include IFN-α, -β, and -ω, and alleviate the effects of viral and bacterial infections in the innate immunity system (4, 5). Type I IFN subtypes-α and -β share common multicomponent, cell surface receptors, and elicit a similar range of biological responses, including antiviral, anti-proliferative (6), and immune modulatory activities.

In this review, we summarize the current knowledge about the role of type I IFN and its inducible genes in the SSc pathogenesis and biomarker development.

## Innate Immunity and SSc

The innate immune system is the first line of host defense against pathogens. It plays an important part in triggering inflammation and promoting development of fibrosis in many organ systems. The dominant cellular components of innate immunity are mainly neutrophils, macrophages, and dendritic cells. These cells sense pathogens and destroy them, followed by secretion of pro-inflammatory chemokines and cytokines to activate T cells and other components of adaptive immune system. There is an increasing evidence for activation of the innate immune system in SSc. Cells involved in the innate immune system are detected at the end organ damage site of SSc (7, 8). Being the first cells in line in the defense against pathogens of any sort, the antigen presenting cells (APCs) are often considered the most influential cell of the innate immune system. The specific nature of these APC and how they contribute to the development of fibrosis is still unclear. The perivascular infiltrates in the non-lesional skin of SSc patients mainly consists of macrophages/monocytes and CD4+ T cells suggesting that the aberrant or dysregulated immune system precedes fibrosis (9–11). Alternatively activated macrophages are present in SSc skin biopsies (8, 12, 13), this type of macrophages are potentially important source of profibrotic cytokines including transforming growth factor β (TGF-β) which contribute to resolving inflammation and promoting wound healing (14). The sub-classification of macrophages into classically activated M1 macrophages and alternatively activated M2 macrophages is also of special interest in SSc because the M1 type is clearly more inflammatory and the M2 type is thought to be more involved in tissue remodeling and profibrotic phenotypes. M2 macrophages highly express several receptors such as hemoglobin scavenger receptor (CD163), class A scavenger receptor (CD204), and mannose receptor (CD206) (15, 16). SSc patients show significantly higher serum soluble CD163 levels, and the number of CD163+ and CD204+ activated M2 macrophages is significantly greater in SSc skin (17, 18). The role of M2 macrophages for the development of fibrosis in SSc is still speculative, further studies are needed to clarify the potential mechanism of M2 macrophages in this disorder.

In this non-specific immune system, mast cells, basophils, and natural killer (NK) T cells play more specialized immune functions. For instance, dermal mast cell number density was significantly higher in diffuse SSc patients than in unaffected controls (19, 20). Electron microscopy (EM) with immunogold labeling in skin biopsy samples revealed that patients with progressive SSc (worsening skin thickening and/or organ function in the year preceding biopsy) had higher number of mast cells. Furthermore, mast cell vesicles containing active TGF-β in patients with SSc showed higher level of degranulation than those from unaffected controls (21). The number of basophils, a circulating counterpart of mast cells was increased in SSc patients. Spontaneous histamine releasability, its reactivity to IgE and response to IL-3 were increased in basophiles from patients with SSc (22). On the other hand, the absolute number and proportion of NK T cells were decreased in patients with SSc which possibly can lead to down-regulation of the normal immune response (23). Altogether, these observations implicate a dysregulated immune system in the pathogenesis of SSc.

In line with those observations, large efforts have been made to find the genetic risk factors for abnormal immune system in SSc. These studies independently replicated genetic risk factors such as *STAT4* (24–26), *BLK* (27–30), *BANK1* (31, 32), Interferon regulatory factor (*IRF*)*-5* (33, 34), *IRF-7*, and *-8* (35–38), and the T cell receptor zeta-chain (*CD247*) (26) which are involved in innate and adaptive immune system.

## Type I IFNs and SSc

Type I IFNs are important key regulators of the innate immune system. They modulate immune cell differentiation and proliferation, as well as inflammatory cytokine production. Recent studies have provided considerable evidence that implicates a dysregulation in type I IFN and IFN-inducible genes in the pathophysiology of autoimmune diseases including SSc (39–43). SSc shares this common characteristic with systemic lupus erythematosus (SLE) (44). Anti-IFNα mAb, sifalimumab, was evaluated in a phase Ia study with an open-label extension of 67 SLE patients with moderately active disease. Sifalimumab caused dose-dependent inhibition of type I IFN-induced mRNAs in whole blood and corresponding changes in related proteins in affected skin. Exploratory analyses showed consistent trends toward improvement in disease activity (44). In a follow-up phase Ib randomized, controlled trial with 161 SLE patients, no statistically significant differences in clinical activity between sifalimumab and placebo were observed. However, when adjusted for excess burst steroids, change in disease activity, and complement levels from baseline showed a positive trend over time (45).

Approximately, half of SSc patients have an increased expression of IFN-regulated genes (termed the “IFN signature”) in their peripheral whole blood cells (46). Recent studies demonstrated activation of type I IFN system was present in SSc sera and plasmacytoid dendritic cells (pDCs) were the main source of IFN-α production (47, 48). Tan et al. first reported a distinct transcript pattern of dysregulated type I IFN-inducible genes in peripheral blood cells (whole blood) from patients with SSc (40). This finding was subsequently confirmed in peripheral blood mononuclear cells (41). The development of SSc has been reported in patients undergoing IFN treatment (49). Furthermore, a randomized, placebo-controlled trial of subcutaneous IFN-α injection in patients with early SSc showed that treatment with IFN-α resulted in worsening lung function and a smaller degree of improvement in skin thickening scores compared to placebo (50). Although the presence of an activated IFN system could be demonstrated, the exact mechanism by which the dysregulated type I IFN signaling contributes to the pathophysiology of fibrosis in SSc is still unknown.

The innate immune system responds rapidly to the presence of certain motifs or patterns that microbes possess commonly such as unmethylated DNA rich in CpG dinucleotides, dsRNA, and bacterial cell wall components via pattern recognition receptors (PRRs) (51). These PRRs are widely expressed on cells of the immune system, as well as endothelial, epithelial, and mesenchymal cells including fibroblasts. Some of the most prominent PRRs are the Toll-like receptors (TLRs). TLRs located on various cellular membranes to sense exogenous and endogenous danger-associated molecular patterns (DAMPs) and pathogen-associated molecular patterns (PAMPs), and play a critical role in innate immune responses. TLR activation triggers production and secretion of several inflammatory cytokines including type I IFNs (52, 53). The TLR family includes both extracellular and endosomal receptors. The first is based on the cell surface like TLR-2, -4, -5, and -6 and recognize patterns found primarily on bacteria, mycobacteria, fungal, and parasitic organisms (54), the latter is located in endosome like TLR-7 and -8. TLR-9 is localized in the endoplasmic reticulum and translocated to the endosome upon response to bacteria DNA. They recognize a wide variety of pathogen components, and all the TLRs except TLR3 signal through the adaptor molecule MyD88, activate and stimulate type I IFN production. Bhattacharyya et al. reported that TLR4 was overexpressed in the skin and lung tissues, as well as explanted skin fibroblasts from patients with SSc (55). Our recent findings revealed that TLR3 expression was upregulated in patients with SSc and IFN-α2 induced an up-regulation of TLR3 in human dermal fibroblasts which is more prominent in SSc patients than in unaffected control subjects (56). These findings are suggesting an important role for TLR activation via type I IFNs in fibroblast biology.

## Interferon Regulatory Factors and SSc

Interferon regulatory factors are best characterized as transcriptional regulators of type I IFNs and IFN-inducible genes and play a pivotal role in regulation of many facets of innate and adaptive immune response (57). This family is composed of nine members: IRF1, IRF2, IRF3, IRF4 (also known as LSIRF, PIP, or ICSAT), IRF5, IRF6, IRF7, IRF8 (also known as ICSBP), and IRF9 (also known as ISGF3γ) (58, 59). As transcriptional factors, each IRF contains a well-conserved DNA-binding domain which is located at the amino terminus and forms a helix-turn-helix-motif. This region recognizes a consensus DNA sequence known as the IFN-stimulated response element (ISRE) in the promoters of targeted genes (59–61). These IRFs coordinate the expression of type I IFNs and type I IFN-inducible genes (57). Several genetic polymorphisms have been associated with SSc in multiple case–control studies and a few family studies (Table 1). Some of these genetic variants are associated with susceptibility for development of SSc, while others act as disease modifiers. Recent genome-wide association studies (GWASs) also confirmed IRFs as genetic susceptibility loci in autoimmune diseases.

**Table 1:** **Polymorphisms in the interferon regulatory factors associated with systemic sclerosis**.

IRF	Chromosome (human)	Expression cells	SSc associated SNPs	Reference
*IRF5*	7q32	B cells; DCs; monocytes	rs2004640; rs2280714; rs10954213; rs3757385	Radstake et al. (26), Dieude et al. (31), Sharif et al. (33)
*IRF7*	11p15.5	B cells; fibroblasts; pDCs; monocytes	rs1131665; rs4963128; rs702966; rs2246614	Carmona et al. (35)
*IRF8*	16q24.1	B cells; macrophages; CD8α + DCs; pDCs; T cells	rs11642873; rs2280381	Gorlova et al. (36), Terao et al. (37), Martin et al. (38)

IRF5 is a transcription factor which induces the transcription of IFN-α and other early pro-inflammatory cytokines (62, 63). *In vitro* experiments have shown that in virus-infected cells, IRF5 is activated by phosphorylation, resulting in nuclear translocation and stimulation of IFN-α (64). Initial analysis of the role of IRF5 in the innate antiviral response utilizing IRF5 mutant mice showed impairment of interleukin-6 (IL-6) and TNF-α production in splenic dendritic cells. IRF5 mutant mice are highly sensitive to viral infection and show lower levels of type I IFN in the serum. IFN production was also impaired in the infected macrophages from IRF5 mutant mice (65, 66). Genetic variants of IRF5 are associated with SSc susceptibility (67–69).

The minor allele of the *IRF5* single-nucleotide polymorphism (SNP) rs4728142 was shown to be predictive of longer survival in the two independent SSc cohorts. The association of this SNP with survival was independent of age at disease onset, disease type, and autoantibody profiles (33). This minor allele was also associated with lower IRF5 transcript expression in monocytes of patients and controls suggesting functional relevance of rs4728142 or it associated SNPs for IRF5 expression.

IRF7 is one of transcription factors involved in IFN signaling pathways which is activated by TLRs TLR3/7/9 or retinoic acid-inducible gene 1 (RIG-1) in response to nucleic acid (both DNA and RNA) immune complexes. Activated IRF7 leads to secretion of a large amount of type I IFN (70). Its expression can potentially be enhanced via a positive feedback loop through IFN receptor and ISGF3 activation, leading to increased IRF7 over-expression and subsequently additional IFNα, transcription (71). IRF7 is essential for the induction of IFN-α/β genes via the virus-activated, MyD88-independent pathway and the TLR-activated, MyD88-dependent pathway (72). Inactive IRF7 resides in the cytoplasm. With pathogenic stimulation, IRF7 is phosphorylated, activated, and translocated into the nucleus, where it forms a transcriptional complex with other co-activators and binds to promoter regions of target genes including IFN-α/-β (73, 74). IRF7 also regulates the pro-inflammatory cytokine IL-6 in pDCs and monocytes (75, 76). The viral induction of MyD88-independent IFN-α/β genes is severely impaired in IRF7 null fibroblasts. Consistently, markedly decreased serum IFN-α level were also observed in IRF7 null mice (72). These studies demonstrated the importance of IRF7 dependent systemic IFN response for the innate immunity. Furthermore, recent genetic studies have established IRF7 as a susceptibility locus in SLE (77–80). Similarly, our group recently reported that a functional variant in the IRF7 exonic region, rs1131665 was associated with SSc (35). These findings support that IRF7 may represent a common risk factor for systemic autoimmune disease processes, including SSc. Microarray studies revealed up-regulation of IRF7 mRNA level in whole peripheral blood cells from SSc patients with early diseases (40). Another independent study showed no statistically significant difference in IRF7 transcript levels in PBMCs of SSc patients compared to controls by quantitative PCR analysis (81). However, patients with late stage disease and a smaller sample size were investigated in this study. Further investigations are needed to determine the contributory role of IRF7 in pathogenesis of SSc.

IRF8 is another immune cell specific IRF family member. It participates in the MyD88-dependent signaling pathway through interaction with TRAF6 (82). IRF8 is required for the induction of Type I IFN genes by viruses and TLR ligands in DCs (83). IRF8 is known to be involved in the development of dendritic cells (84). IRF8 also promotes B cell differentiation (85). Recently, the *IRF8* SNP, rs11642873 was identified as a risk factor for limited and anti-centromere positive SSc patients in a large GWAS follow-up study conducted in European and North-American cohorts (36). Another independent study identified rs2280381 polymorphism in IRF8 as a susceptibility locus of SSc in the Japanese population (37). The association of *IRF8* genetic variants with SSc supports possible involvement of B cells and dendritic cells in the development of SSc. However, the role and importance of B cells or dendritic cells in the fibrotic component of SSc has not been well established (86–88).

Further fine-mapping and functional studies are crucial for elucidating the role of genetic variants in the IRFs in the pathogenesis of SSc.

## Interferon Inducible Cytokines and Chemokines in Scleroderma

Interleukin-6 is one of the most prominent cytokines activated by IFN pathway. It is involved in the pathogenesis of many immune-mediated diseases including SSc (89–91). IL-6 is a classic inflammatory cytokine produced by various cells and involved in B cell differentiation, induction of acute phase proteins in liver cells, proliferation, and differentiation of T cells (92, 93). By binding to the IL-6 receptor (IL-6R)-α chain and the signal transducing component gp130 (CD130), pleiotropic IL-6 activates downstream signaling mediated by STAT1 or STAT3 through tyrosine phosphorylation. Previous studies have shown that IL-6 plays an important role in the initiation and promotion of fibrosis (94, 95). Production of IL-6 and soluble IL-6R by cultured peripheral blood mononuclear cells were significantly higher in patients with SSc and soluble IL-6R levels significantly correlated with the severity of pulmonary fibrosis in patients with SSc (96). Serum IL-6 levels might be predictive of disease progression in Interstitial lung disease associated with SSc (97). IL-6 shifts T cells from regulatory response to pathogenic Th17 response (98), and promotes the differentiation of CD4+ cells to a profibrotic Th2 type while suppressing Th1 differentiation (99). IL-6 stimulation induces increased collagen production in dermal fibroblasts (100, 101). These studies demonstrate that IL-6 is involved in the pathogenesis of SSc and may contribute to progression of fibrosis and disease severity in SSc.

A combined score of the plasma IFN-inducible chemokines, IFNγ-inducible protein 10 (IP-10/CXCL10), and IFN-inducible T cell α chemoattractant (I-TAC/CXCL11) highly correlated with the IFN gene expression signature in SSc patients in the Genetics versus Environment in Scleroderma Outcome Study (GENISOS) cohort study (102). As expected, SSc patients had higher IFN-inducible chemokine scores than age-, gender-, and ethnicity-matched controls. Among 266 SSc patients, the IFN-inducible chemokine score was associated with presence of anti-U1 RNP antibodies while patients with anti-RNA polymerase III antibodies had lower levels of this chemokine score. The lower IFN chemokine levels in patients with anti-RNA polymerase III antibodies might be of important biological significance because these antibodies are associated with presence of diffuse cutaneous involvement and absence of severe interstitial lung disease. The IFN-inducible chemokine score was not associated with disease duration, disease type, or other auto-antibodies. The chemokine score correlated positively with the concomitantly obtained scores on the Medsger Severity Index for muscle, skin, and lung involvement, as well as creatine kinase levels in SSc. There was also a negative correlation with forced vital capacity and diffusing capacity for carbon monoxide. These results support the aforementioned findings that the IFN activation is associated with the more severe form of SSc. There was no significant change observed in the IFN-inducible chemokine score over time in SSc patients. The fact that the IFN chemokine score did not show a consistent trend of change and that it was not associated with disease duration at the baseline visit indicates that the IFN signature is a stable marker for the more severe subtype of disease rather than a time-dependent immune dysregulation that improves after the initial phase of SSc (102).

## Conclusion

There are many distinct immunological and molecular mechanisms that can contribute to pathogenesis and progression of SSc. Dysregulated innate and adaptive immune responses are major contributors to fibrosis and disease severity of SSc. This review summarized a possible role of type I IFN and IFN-inducible genes in pathogenesis of SSc, and provides support for a link between type I IFN and fibrosis in SSc. Potential role of type I IFN or IFN-inducible genes as treatment targets or biomarkers in SSc need to be further explored. A better understanding of the relationship between type I IFN and fibrosis could bring us closer to the ultimate goal of reversing or slowing the fibrotic process and regenerating the normal end organ tissue in SSc.

## Conflict of Interest Statement

The authors declare that the research was conducted in the absence of any commercial or financial relationships that could be construed as a potential conflict of interest.
